# The Decreasing Trend in Dietary Glycaemic Index and Glycaemic Load in Australian Children and Adolescents between 1995 and 2012

**DOI:** 10.3390/nu10091312

**Published:** 2018-09-16

**Authors:** Chris Ho Ching Yeung, Devina Tri Lestrai Kusnadi, Alan Winston Barclay, Jennie Cecile Brand-Miller, Jimmy Chun Yu Louie

**Affiliations:** 1 School of Biological Sciences, Faculty of Science, The University of Hong Kong, Pokfulam, Hong Kong, China; cyhc321@gmail.com; 2 Charles Perkins Centre and School of Life and Environmental Sciences, The University of Sydney, Sydney 2006, NSW, Australia; dkus5312@uni.sydney.edu.au (D.T.L.K.); jennie.brandmiller@sydney.edu.au (J.C.B.-M.); 3 Private Practice, Sydney, NSW, Australia; awbarclay@optusnet.com.au

**Keywords:** glycaemic index, glycaemic load, Australian children and adolescents, national nutrition surveys, trend analysis

## Abstract

This study aims to examine whether there were changes between 1995–2012 in the dietary glycaemic index (dGI) and glycaemic load (dGL) in Australian children (<16 years) according to three national surveys in 1995 (1995NS), 2007 (2007NS), and 2011–2012 (2012NS). Glycaemic index (GI) values of foods were assigned using published methodology. Plausible 24-h recall data from the 1995NS, 2007NS and 2012NS (weighted *n* = 2475, 4373 and 1691 respectively) were compared for differences in dGI and dGL, and the contribution to dGL from different foods using one-way ANOVA with Bonferroni post hoc comparisons and linear regression. Decreasing trends across surveys were found in dGI and dGL (*p* < 0.001). Between 1995 and 2012, dGI and dGL per Megajoule (MJ) dropped by 2% and 6% respectively. The per capita dGL contribution from breads and bread rolls, fruit and vegetable juices, sweetened beverages and potatoes showed strong decreasing trends (*R*^2^ *>* 0.7). Our findings suggest that dGI and dGL of Australian youths declined between 1995 to 2012, which may be due to increased awareness of the GI concept and healthy diet, widened food choices and immigrants with diverse dietary habits. This may lower the future risks of chronic degenerative diseases in Australian youths.

## 1. Introduction

The relationship between dietary carbohydrates, postprandial glycaemia and health outcomes remains controversial. One measure of carbohydrate quality, the glycaemic index (GI), was first introduced in 1981 as a metric describing the extent to which blood glucose is raised by available carbohydrates in different foods [1]. By definition, low GI foods have GI values less than 55 (e.g., most dairy products), moderate GI from 56 to 69 (e.g., rice noodles, honey) and high GI at or above 70 (e.g., refined carbohydrates such as white rice) [2]. A lower GI indicates that the carbohydrates in a given food has a lower effect on postprandial blood glucose and possibly insulin responses. In contrast, the carbohydrates in high GI foods will lead to a greater surge of postprandial blood glucose level [3]. Since GI relates only to the quality of the carbohydrate, the concept of glycaemic load (GL) was proposed by Salmeron et al. [4] to represent both the quality and quantity (portion size) of carbohydrates. GL has been shown to be superior to the absolute amount of carbohydrate alone in predicting postprandial glycaemia in the context of both single foods and mixed meals [5]. 

Through the GI symbol program, popular press, television, advertising and the internet, Australian consumers have been exposed to the GI concept for over two decades [6,7,8,9]. Over the same timeframe, there have been changes in food availability, macronutrient distribution [10], food commodity groups [11] and health education in schools [12]. It is therefore reasonable to suggest changes in carbohydrate quality may have also taken place. 

Previous studies in adults have reported association between high GI/GL intake with higher risk of diabetes, cancer, central obesity and higher BMI [13,14,15,16,17]. Studies in adolescents showed a positive association between GI/GL with blood pressure and risk of overweight/obesity [18,19]. An analysis of longitudinal trends in dGI/dGL of children can provide insights into education and policy changes needed to encourage healthier sources of carbohydrate energy which may help reduce the risk of chronic degenerative diseases on a population scale. 

Our group have previously investigated the cross-sectional dietary data of Australian adolescents in 2007 and 2012 and the changes in healthy and unhealthy food intakes between 1995 and 2007, where we showed that Australian youths were generally had a healthier diet in 2007 than in 1995, and they appeared to have a lower dGI than European children in 2011–2012 [20,21,22]. However, to our knowledge, no study has explored dGI/dGL trends over time in children and adolescents. Using three national surveys can also provide us with additional information on the trends compared with only two years of data available in our previous analyses. The objectives of this study were to examine the current status and trends in dGI, dGL, and the major contributory food groups in Australian children aged 2 to 16 years according to national dietary surveys available to date conducted in 1995, 2007 and 2012. We hypothesized there may have been a decrease in dGI/dGL in Australian children and adolescents over time due to increased awareness of the GI concept, changes in food availability, macronutrient distribution and health education.

## 2. Materials and Methods 

### 2.1. Data Used

#### 2.1.1. 1995 Australian National Nutrition Survey (1995NS)

The 1995NS was conducted by the Australian Bureau of Statistics (ABS) and the Department of Health together with the 1995 National Health Survey [23]. Data collection was conducted seven days a week between February 1995 and March 1996 [24]. Information about food and beverage intake, usual frequency of intake, eating habit, attitudes and physical measurements of Australians population aged two year-old or above was collected (*n* = 13,858) with a 61.4% response rate. Participants were selected randomly from dwellings in different State and Territories. Dietary intake was assessed using a single 24-h recall, and a subset of the sample (76.2% of those aged 12 and over) also completed a food frequency questionnaire (FFQ) [23]. However the FFQ data were not used in the current analysis. Children aged 15 to 16 years provided their own dietary recall while parents, guardians or close relatives were responsible for providing the information for children aged 14 years or below [25,26]. Among all the participants in 1995NS, 2729 (19.7%) were aged between 2 and 16 inclusively.

#### 2.1.2. 2007 Australian National Children’s Nutrition and Physical Activity Survey (2007NS)

Details and cross-sectional dGI/dGL data of the 2007NS were previously described [21]. In brief, information on dietary intake, physical activity level (PAL) and demographic characteristics of randomly selected children and adolescents aged 2 to 16 years (*n* = 4837) from all Australian states and territories were collected with a response rate of 40%. Data collection occurred between February 2007 and August 2007. Dietary data were collected using a 24-h recall during face-to face home visits, and the majority of the sample (*n* = 4658) completed a second 24-h recall during a telephone interview conducted 7–21 days after the home visit. The first and second interview were conducted on different day types (weekdays/weekends) when possible. Care-givers provided dietary recall information for children aged 2 to 8 years while children aged nine years or above provided their own dietary recall [25,27,28].

#### 2.1.3. 2011-12 National Nutrition and Physical Activity Survey (2012NS)

Details and cross-sectional dGI/dGL data of the 2012NS have been previously described by our group [20]. In brief, respondents were randomly selected from 9500 private dwellings across Australia except for very remote areas and some non-private dwellings, e.g., hotels and hospitals. Dietary and physical activity information of the Australian population aged two years or above (*n* = 12,153) were collected from May 2011 to June 2012 from 12,366 dwellings with a 77% response rate. Data were mainly collected on Monday to Saturday depending on respondents’ availability and were occasionally collected on Sunday when specifically requested by respondents. Dietary data were collected using a 24-h recall during face-to-face home visits, and a second 24-h recall during a telephone interview was collected in ~60% of subjects at least eight days after the home visit. Adults were responsible for providing full food recall and responding to the physical activity questions for children aged 2 to 5 years, while children aged 6 to 8 years can assist in the recall. Children aged 9 to 11 years were interviewed directly with adults assisting, and children aged 12 to 14 years answered the questions themselves with adults in the same room. Those aged 15 to 17 years were interviewed personally with parental consent [26,29,30,31,32]. Among all the participants in 2012NS, 2548 (20.9%) were aged between 2 and 16 years inclusively.

**Figure A1:**
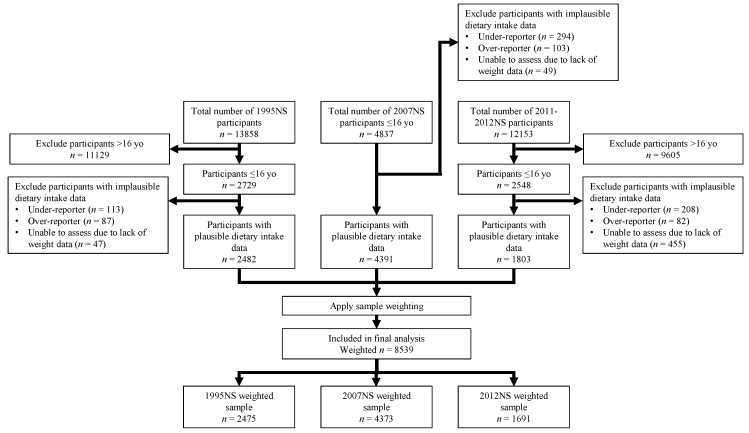
Flow of participants.

To allow direct comparison between surveys, only data from the first 24-h recall in the three surveys were used due to the low response rate of the second 24-h recall in the 1995NS; and only data from subjects aged 2 to 16 years were used as the 2007NS did not include adolescents aged 17 to 18 years. Data from the three surveys were combined into one dataset for analysis. A flow-of-participants diagram is available as Figure A1.

### 2.2. Dietary Glycemic Load and Dietary Glycemic Index Calculation

GI values were assigned to foods in AUSNUT1999 [33] (for 1995NS), AUSNUT2007 [34] (for 2007NS) and AUSNUT2011-2013 [35] (for 2012NS) food composition tables as previously described [36]. These values were then matched with the food intake of the respondents of the three surveys. GL of each food item was calculated by multiplying the GI by the available carbohydrates in a serving of the food. dGL was the sum of GL from all the foods reported in the 24-h recall while dGI was calculated by dividing an individual’s dGL by the total available carbohydrates intake of that person, expressed as a percentage. 

### 2.3. Re-Coding and Classification of Food Groups in the Three Surveys

Food groups in the three AUSNUT databases were recoded into similar food groups for comparability purposes as described previously [37]. The final recoding table is shown in the Table A1.

### 2.4. Data Cleaning

Data were cleaned as previously described [37]. A default PAL of 1.55 was assigned to participants without PAL data, which included participants aged ≤8 years in the 2007NS (*n* = 2494), and all participants in 1995NS and 2012NS. For children aged 9 to 16 in 2007NS, PAL was calculated by activity data collected using a validated 24-h recall [30]. All participants with energy intake to basal metabolic rate ratio (EI:BMR) outside the 95%CI calculated based on the Goldberg cut-off for specific PAL method were consider to have extremely implausible food intake. Value of CV_wB_, CV_tP_ and CV_wEI_ used in the calculation were set at 8.5, 15 and 23 respectively according to previous studies [38,39]. Using PAL of 1.55 as an example, the cut-off point was calculated to be 0.87–2.75. For 1995NS, 2007NS and 2012NS, 200 out of 2729 (73%, with 113 extreme under- and 87 extreme over-reporters), 397 out of 4837 (5.1%, with 294 extreme under- and 103 extreme over-reporters) and 745 out of 2548 (31.4%, with 208 extreme under- and 82 extreme over-reporters) were excluded from the analysis. A further 551 (*n* = 47, 49 and 455 for 1995NS, 2007NS and 2012NS respectively) respondents who had no weight data, which disallowed the computation of the energy intake to basal metabolic rate ratio (EI:BMR), were also excluded. Sensitivity analyses were performed where data of all subjects including under- and over-reporters were used (Table A2, Table A3, Table A4, Table A5 and Table A6), and no material difference in the findings and conclusions were observed. Thus in this study we excluded under- and over-reporters to obtain more accurate population estimates.

### 2.5. Statistical Analysis

This secondary analysis was registered at anzctr.org.au (reference number: ACTRN12617000992303). Data were weighted to adjust for over- and/or under-sampling in terms of age, gender and region which may occur due to errors related to scope and coverage in random sampling and non-response bias, so as to represent Australian children and adolescents. The sample weightings supplied with the survey datasets were readjusted to account for the exclusion of extreme under- and over-reporters. Comparisons of dGI, dGL and percentage energy from different nutrients between the three surveys were done using one-way ANOVA. The same was used for the comparison of the top contributors of dGL food groups, both *per capita* and per consumer. Percentage of consumers were also stated in the per consumer table. Food groups with less than 10 consumers were excluded as they were considered non-representative. Results were presented as mean and standard deviation (SD) and were ranked in the GL contributors table. Bonferroni post hoc analysis was conducted to test for difference between any two surveys. Linear regression on the median BMI, GI, GL, different nutrients and per consumer top GL contributing food groups were also done to examine whether linear trends exist across the three surveys. Median was used as it is less likely to be affected by outliers, or skewed by zero (i.e., non-consumers). Mean instead of median was used for the linear regression on *per capita* top GL contributing food groups as the majority of the medians were zero. A linear regression model with an *R*^2^ > 0.7 was considered a good fit. Multiple linear regression was performed using intake of energy and the top 20 GL contributing food groups as independent variables to describe the inter-individual variations in dGI and dGL. All statistical analyses were performed using SPSS version 23.0 (IBM Co. Ltd, Armonk, NY, USA), assuming normal distribution [30,40]. Due to the large number of comparisons made, a *p* value of 0.001 was set to indicate statistical significance [41].

## 3. Results

### 3.1. Characteristics of Participants

The analysis included a weighted sample of 8539 participants with 2475 from 1995NS, 4373 from 2007NS and 1691 from 2012NS as shown in Table 1. The three surveys had similar male:female ratios; however the 2012NS had more respondents aged 9 to 13 years and fewer respondents aged 14 to 16 years when compared with the other two surveys. It also included more non-Australian born respondents than the two earlier surveys (*p* < 0.001). A higher proportion of excluded participants were from the 14 to 16 years age group (30.3% vs. 19.6%), while a lower proportion were from 2 to 3 years (16.9 vs. 20.4%) and 4 to 8 years age groups (24.1% vs. 31.0%) when compared with included participants (*p* < 0.001). There were no other significant differences between excluded vs. included participants in terms of sex, and country of birth.

### 3.2. Trends in BMI, GI, GL, Energy, Macronutrients and Fiber Intake

Table 2 shows the mean BMI, dGI, dGL and the daily percentage energy contribution from selected macronutrients across the three surveys. No significant difference or trend was found in the mean BMI across the surveys. General decreasing trends were found in the median dGI, dGL, and GL per MJ, but the magnitude was usually small. Significant decreasing trends were also found in the median energy intake and percentage energy from sugars (*p* < 0.001). On the other hand, median percentage energy from starch had an increasing trend.

### 3.3. GL Contribution, Per Capita

Table 3 shows the top GL contributing food groups in the three surveys. Breads were found to be the highest in all three surveys (18.1% in 1995, 15.6% in 2007, 15.0% in 2012). Ready to eat breakfast cereal (9.2%, 9.2%, 6.7%) and juices (9.9%, 6.5%, 5.5%) were in the top five in each case. Potatoes (7.5%, 5.7%, 4.3%) and sweetened beverage (5.9%, 3.8%, 3.9%) were in the top five in 1995 but the ranking dropped to 6th and 7th in 2012 respectively. Although the ranking remained high, their contributing percentage dropped. Cereal-based dishes ranked 6th and 9th in 1995 and 2007 respectively but then rose to 2nd in 2012 (3.9%, 3.6%, 10.0%). The rank of cow’s milk dropped from 7th in 1995 and 2007 to 11th in 2012, while frozen milk products dropped from 11th to 21st then 19th. The ranking of starches (3.2%, 4.1%, 4.6%) rose from 9th to 6th and 5th, while savory biscuits (1.5%, 2.7%, 3.2%) rose from 18th to 12th and 10th. Fancy breads also went up from 20th to 13th and 12th in the same period.

The results of linear regression showed that there were decreasing trends (*p*_trend_ < 0.001 and *R*^2^ > 0.7) in the mean percentage GL contribution of breads and bread rolls, fruit and vegetable juices, breakfast cereals (ready to eat), potatoes, sweetened beverages, sugar, honey and syrup, frozen milk products, and milk and milk product-based dishes. On the other hand, there were increasing trends (*p*_trend_ < 0.001 and *R*^2^ > 0.7) for flours, cereal and starches, savory biscuits, fancy breads, pome fruits, tropical and subtropical fruits, poultry-based dishes, and cereal-, fruit-, nut-, and seed-bars groups. For the rest of the food groups, while the linear trend was statistically significant, the *R*^2^ indicated that year of survey was not a good predictor of change in mean percentage dGL contribution.

### 3.4. GL Contribution, Per Consumer

Per consumer percentage GL contribution is shown in Table 4. Flours, cereals and starches ranked the 1st in all three surveys (22.8%, 22.2%, 27.3%). Breads ranked 2nd in 1995 and 2007 and 3rd in 2012 (21.4%, 20.2%, 20.3%). Cereal-based dishes ranked 3rd in 1995, dropped to 6th in 2007, then rose to 2nd in 2012 (16.6%, 15.2%, 23.7%). Hot porridge (16.4%, 17.5%, 15.5%) and ready-to-eat (15.8%, 17.2%, 15.3%) breakfast cereals were among top 5 in 1995 and 2007 and then dropped to 7th and 8th respectively in 2012. Rank of sweetened beverage dropped from 6th to 11th and then 16th (15.4%, 12.2%, 10.5%). Pastas (14.1%, 16.0%, 16.9%) went up from 9th to 5th and to 6th and cake-type desserts (14.0%, 14.1%, 18.5%) went up from 10th to 8th and then 4th. The ranking for fruit and vegetable juices (13.9%, 10.9%, 11.2%) dropped from 11th in 1995 to 14th in 2007 and 2012.

Linear regression showed that there were decreasing trends (all *p*_trend_ < 0.001 and *R*^2^ > 0.7) in breads, potatoes, batter-based products, sweetened beverages, pretzels and other snacks, and fruit and vegetable juices from 1995 to 2012. On the other hand, increasing trends (all *p*_trend_ < 0.001 and *R*^2^ > 0.7) were found in pastas, pastries, savory biscuits, fruit combinations, chocolates, potato snacks, cereal-, fruit-, nut-, seed- bars and tropical fruits. For the rest of the food groups, either the model was not statistically significant or the *R*^2^ values were low, which suggested that year of survey was not a good predictor of median change in dGL contribution.

### 3.5. Inter-Individual Variations in Dietary GI and GL

The contribution of the top 20 GL food groups in both surveys to the inter-individual variation of dGI and dGL is presented in Table 5, Table 6 and Table 7. Overall, there were decreases over time in the inter-individual variation in dGI (*R*^2^: 0.441, 0.372 and 0.351 for 1995NS, 2007NS and 2012NS respectively) and dGL (*R*^2^: 0.888, 0.862 and 0.846 for 1995NS, 2007NS and 2012NS respectively) explained by these food groups.

## 4. Discussion

In this analysis, we report downward trends in the mean dGI and dGL in Australian children between 1995 and 2012. Over this timeframe, major carbohydrate food groups made a smaller contribution to overall dGL. Energy-dense nutrient-poor food groups such as sugar-sweetened beverages (SSB) and juice declined, while starch energy and cereals-based products, and savory biscuits increased.

Few observational studies [42,43] had examined longitudinal trends in dGI and dGL, or changes in the contribution of different food groups. To our knowledge, our study is the first utilizing a nationally representative sample to address this evidence gap. Results from the DONALD study provide interesting comparisons. Among German children in the DONALD study who were 7 to 8 years old in the 1990, 1996 and 2002 cross-sections, dGI increased from 55.1 to 56.0 then 56.5 in the 12 year period, while GL per MJ had increased from 16.7 to 17.5 then plateaued [42]. This contrasts with our finding that Australian children and adolescents had a lower dGL over a 17-year period, which could be largely explained by the diverging trends in carbohydrate food choices. The non-representative sample of high socio-economic status participants in the DONALD study may have different dietary habits, such as higher GI and GL diet, which may not represent the general population and led to the discrepancies.

As expected, breads made the highest contribution to dGL in the *per capita* analysis and ranked among the top three on a per consumer basis across all three surveys. Although there are low GI specialty breads on the market, most types have a relatively high GI [2,44]. A previous analysis of the 2012NS reported that two in three Australians consumed breads on the survey day [29]. Nonetheless, the contribution of breads to dGL fell progressively from 18% to 16% to 15% on a *per capita* basis in 1995, 2007 and 2012 respectively. One explanation may be the increasing popularity of branded whole grain breads that are specifically marketed on the basis of their low GI values. This is supported by our previous study [22] comparing the core food intake of Australian children between 1995 and 2007, which showed a decrease in *per capita* white bread intake from 61 to 41 g in 2 to 16 years old children, and an increase in wholegrain bread from 15 to 23 g.

The mean dGL contribution of cereal-based dishes increased markedly from 1995 to 2012. Their contribution to dGL increased from 4% to 10% *per capita* and from 17% to 24% per consumer. Other than an increase in consumption, the increases may also be influenced by changes in data collection and coding methods in the 2012NS [31]. Modifications were made to the AUSNUT2011-2013 classification system due to the changes in food supply across the years and to ensure sufficient details were captured to meet the needs of future users [45]. Thus some of the foods in the cereal-based dishes group in 2012NS were not included in AUSNUT1999 and AUSNUT2007, e.g., flavored rice and dumplings [46]. Similarly, in 1995NS and 2007NS, mixed foods such as burgers were split into individual ingredients (bun, patty and fillings), while in 2012NS, these foods were coded and reported as a single mixed food [30,31]. A 150 g hot dog which belonged to the cereal-based dishes group in 2012NS may have been separated into an 80 g sausage in the meat group with a 70 g bread roll in the cereal products group in 1995NS and 2007NS. As a result, it is possible that more foods were included in the cereal-based group in 2012NS, thereby increasing the GL contribution of this group.

The top five food groups *per capita* in 1995 (breads and bread rolls, fruit and vegetable juices, breakfast cereals (ready to eat), potatoes and SSB), had all fallen by 2012. On the other hand, the GL contribution of cereal-based dishes, cakes, flours, cereals and starches, and fruits rose. The decreased consumption of these carbohydrate-rich foods (breads and bread rolls, breakfast cereals (ready to eat) and potatoes) could be due to the increased popularity of low GI diet since around 2002, and low carbohydrate diets being introduced in Australia around 2004 [6,47]. Media coverage of the high amount of sugars in drinks, possible health risks brought by high fruit juice consumption, as well as the banning of SSBs in public schools in Australian states since 2007 may also have increased the awareness towards these drinks, and contributed to the decreasing trend in dGL contributed by SSBs and fruit juices [48,49,50], although SSB and fruit juices were still major food choices for certain children, contributing >10% to dGL on a per consumer basis across all three surveys. While fruit juice is rich in vitamins and phenolic antioxidants, its consumption as a replacement for whole fruits is currently discouraged [51].

From the multiple linear regression analysis, the decreasing *R*^2^ indicates the intake of the top 20 food groups contributing to GL explained less and less of the inter-individual variations in dGI and GL. This finding is consistent with our previous analysis in Australian adults [37]. Overall, our findings suggest that Australian children and adolescents are consuming a more diverse diet, or that their food intake patterns have changed in recent years. This could be due to increased awareness of healthy diets from the media, as well as the aforementioned regulations on SSBs which may have led to changes in the food and drinks consumption patterns. We performed post hoc analyses on changes in absolute carbohydrate intakes across the three surveys and found carbohydrate intake dropped by ~9% in this period (data not shown). In addition, data from the ABS showed household expenditure on certain foods such as breads, cakes and cereals have dropped, while that for meals out and takeaway foods had increased from 1998/99 to 2009/10 [52]. This may indicate that Australian youths may have a wider variety of food choices, hence developing a more diverse diet. On the other hand, migrants from South Asian and South-East Asian countries have contributed markedly to Australia’s population growth and undoubtedly to changing food trends. In June 2013, 6.4 million people from a total population of 23 million were overseas-born migrants. Those from China, India, Vietnam and other countries often maintain traditional eating habits and influence local food customs [53].

Our analyses showed decreasing trends in dGI and dGL of Australian youths from 1995 to 2012. The drop in dGI and dGL may provide a positive impact to the health of Australian youths, such as lower blood pressure and risk of overweight/obesity [18,19]. The risk of chronic degenerative diseases such as type 2 diabetes and cardiovascular diseases could also be seen if the low GI/GL dietary habit could persist to adulthood [13,14,15,16,17,54]. While the effect on some diseases at an individual level may not be huge, the population effect could be significant.

The strengths of this study include the use of representative national dietary data collected and analyzed using similar methods in all three surveys. The longitudinal statistical comparisons allowed us to distinguish statistically significant changes from apparent changes. GI values were assigned using a published method [36] which increases the reliability of the findings. While under-reporting is a likely component of all national dietary surveys [55], we applied a validated method to exclude extreme under- and over-reporters to improve the precision of population estimates [38]. Results of the sensitivity analyses indicated that exclusion of extreme mis-reporters did not bias the results.

Several limitations must also be considered. The 1995NS and 2012NS collected data throughout the whole year, while the 2007 survey was conducted during fall and winter months only. This may have led to differences in reported consumption of certain seasonal foods, particularly ice-cream and SSB. Second, children provided their own dietary recall at a younger age in the 2007NS and 2012NS, and different visual-aids were used for estimating portion size which may have impacted the accuracy of reporting. Finally, we utilized data from only 1 × 24-h recall as the response rate for the second recall in the 1995NS was low, although the use of 1 × 24-h recall is adequate in generating accurate population means [56].

## 5. Conclusions

Our findings suggest that dGI and dGL of Australian children and adolescents declined between 1995 to 2012. There were qualitative changes in the foods contributing the most to overall dGL. Breads, fruit juices, SSBs and potatoes showed decreasing trends on both a *per capita* and per consumer basis. These trends may have been influenced by increased awareness of benefits of healthy sources of carbohydrates, or changes in eating habits of the population, possibly as a result of increased knowledge of healthy diets and the GI concept, more diverse food choices, and immigration from Asian countries.

## Figures and Tables

**Table 1:** Demographic characteristics of included participants.

	1995NS	2007NS	2012NS	*p* Value ^1^
*n* ^2^	2475	4373	1691	-
Male (%)	51.6	51.7	51.5	0.984
Age groups (%) ^3^				
2–3 years	13.4	13.2	12.7	0.181
4–8 years	34.3	34.4	32.6	
9–13 years	33.7	33.4	37.3	
14–16 years	18.6	19.1	17.4	
Country of birth (%)				
Australia	94.3	92.5	90.2	<0.001
Others	5.7	7.5	9.8	

^1^ *p* value tested by chi-squared test for differences between the three surveys; ^2^ The sample were weighted and extreme under- and over-reporters were excluded; ^3^ Age groups as defined in the 2007 Children’s Survey; due to rounding to 1 decimal place the % may add up to more than 100%.

**Table 2:** Mean ± SD daily glycemic index, glycemic load and intake of macronutrients of respondents of the three surveys.

	1995NS	2007NS	2012NS	*p*_trend_^1^
BMI (kg/m^2^) ^2^	18.3 ± 3.4	18.5 ± 3.6	18.6 ± 3.5	0.033
Dietary GI	56.7 ± 5.1	54.2 ± 5.6 ^3^	55.4 ± 5.3 ^3,4^	<0.001
Dietary GL	153.0 ± 58.6	141.0 ± 54.5 ^3^	135.6 ± 50.0 ^3^	<0.001
Dietary GL (g/MJ)	17.9 ± 3.3	16.7 ± 3.3 ^3^	16.8 ± 3.3 ^3^	<0.001
Energy (kJ)	8590 ± 2980	8500 ± 2890	8100 ± 2640 ^3,4^	<0.001
Energy from fat (%)	33.2 ± 6.6	30.6 ± 6.9 ^3^	31.2 ± 6.9 ^3^	<0.001
Energy from saturated fat (%)	14.6 ± 4.0	13.7 ± 4.1 ^3^	13.2 ± 4.0 ^3,4^	<0.001
Energy from protein (%)	14.4 ± 3.7	16.4 ± 4.5 ^3^	15.9 ± 4.2 ^3,4^	<0.001
Energy from carbohydrates (%)	53.6 ± 8.2	52.2 ± 8.1 ^3^	51.5 ± 7.9 ^3^	<0.001
Energy from sugars (%)	26.4 ± 8.9	25.6 ± 7.9 ^3^	22.9 ± 7.6 ^3,4^	<0.001
Energy from starch (%)	25.3 ± 7.1	26.0 ± 7.4 ^3^	26.9 ± 7.5 ^3,4^	<0.001
Fibre density (g/MJ)	2.2 ± 0.8	2.5 ± 0.9 ^3^	2.6 ± 1.0 ^3^	<0.001

^1^ *p*_trend_ from linear regression test for trends in median of the three surveys; ^2^ *n* = 2435, 4373 and 1684 due to missing values; ^3^ *p* < 0.001 compared with 1995NS; ^4^ *p* < 0.001 compared with 2007NS.

**Table 3:** Per capita mean ± SD comparison of the highest contributors to glycemic load in the three surveys.

Food Groups	1995NS		2007NS		2012NS		*β* ± SE ^4^	*R*^2^	*p*_trend_^1^
	Rank	Mean ± SD	Rank	Mean ± SD	Rank	Mean ± SD			
Bread and bread rolls	1	18.1 ± 13.6	1	15.6 ± 13.4 ^2^	1	15.0 ± 12.9	−0.197 ± 0.000	0.998	<0.001
Fruits and vegetables juices	2	9.9 ± 10.5	3	6.5 ± 8.3 ^2^	4	5.5 ± 8.1 ^2,3^	−0.279 ± 0.000	1.000	<0.001
Breakfast cereals (ready to eat)	3	9.2 ± 10.5	2	9.2 ± 11.2	3	6.7 ± 9.6 ^2,3^	−0.086 ± 0.001	0.304	<0.001
Potatoes	4	7.5 ± 10.5	4	5.7 ± 9.0	6	4.3 ± 8.5	−0.178 ± 0.000	0.942	<0.001
Sweetened beverages	5	5.9 ± 10.3	8	3.8 ± 7.6 ^2^	7	3.9 ± 8.1 ^2^	−0.141 ± 0.000	0.942	<0.001
Cereal based dishes	6	3.9 ± 9.1	9	3.4 ± 8.0	2	10.0 ± 15.0 ^2,3^	0.201 ± 0.004	0.238	<0.001
Dairy milk	7	3.2 ± 3.6	7	3.8 ± 4.1 ^2^	11	3.0 ± 4.0 ^3^	0.017 ± 0.001	0.108	<0.001
Cake-type dessert	8	3.2 ± 7.7	10	3.1 ± 7.3	8	3.8 ± 9.3	0.016 ± 0.001	0.106	<0.001
Flours, cereals and starches	9	3.2 ± 9.8	6	4.1 ± 10.9	5	4.6 ± 12.3 ^2^	0.082 ± 0.000	0.959	<0.001
Sweet biscuits	10	2.8 ± 5.3	11	2.7 ± 5.4	9	3.4 ± 6.3 ^3^	0.011 ± 0.000	0.065	<0.001
Frozen milk products	11	2.4 ± 4.6	21	1.6 ± 3.6 ^2^	19	1.6 ± 3.7 ^2^	−0.057 ± 0.000	0.951	<0.001
Pastas	12	2.2 ± 6.2	5	4.2 ± 8.5 ^2^	16	1.9 ± 6.2 ^3^	0.062 ± 0.002	0.125	<0.001
Sugar, honey and syrups	13	2.1 ± 3.8	19	1.8 ± 3.7	18	1.6 ± 3.9 ^2^	−0.028 ± 0.000	0.929	<0.001
Pome fruit	14	2.0 ± 3.6	15	2.3 ± 3.8	13	2.7 ± 4.3 ^2,3^	0.033 ± 0.000	0.742	<0.001
Pastries	15	2.0 ± 5.4	18	2.0 ± 5.6	15	1.9 ± 5.6	−0.002 ± 0.000	0.097	<0.001
Other confectionery	16	1.9 ± 5.6	14	2.6 ± 7.3 ^2^	23	1.3 ± 3.8 ^3^	0.003 ± 0.001	0.002	<0.001
Chocolates	17	1.7 ± 4.3	16	2.1 ± 5.5	21	1.4 ± 4.5 ^3^	0.004 ± 0.000	0.008	<0.001
Savory biscuits	18	1.5 ± 4.1	12	2.7 ± 5.9 ^2^	10	3.2 ± 7.3 ^2^	0.101 ± 0.000	0.989	<0.001
Tropical and subtropical fruit	19	1.4 ± 3.6	17	2.0 ± 4.3 ^2^	14	2.0 ± 4.6 ^2^	0.041 ± 0.000	0.945	<0.001
Fancy breads	20	1.3 ± 4.5	13	2.7 ± 7.0 ^2^	12	2.9 ± 7.7 ^2^	0.110 ± 0.000	0.973	<0.001
Potato snacks	21	1.1 ± 2.9	25	1.2 ± 3.6	22	1.3 ± 4.1	0.008 ± 0.000	0.588	<0.001
Confectionery based dishes	22	1.0 ± 3.6	28	0.8 ± 3.1	28	0.9 ± 3.0	0.003 ± 0.001	0.002	<0.001
Batter-based product	23	1.0 ± 4.5	20	1.7 ± 5.6 ^2^	27	1.0 ± 4.0 ^3^	0.022 ± 0.001	0.157	<0.001
Cereal-, fruit-, nut-,seed-bars	24	1.0 ± 2.6	22	1.4 ± 4.0	17	1.8 ± 4.5	0.043 ± 0.000	0.878	<0.001
Extruded snacks	25	0.8 ± 3.4	44	0.2 ± 1.7 ^2^	33	0.6 ± 3.2 ^3^	−0.033 ± 0.000	0.523	<0.001
Milk and milk products based dishes	26	0.8 ± 2.8	35	0.5 ± 2.3 ^2^	39	0.3 ± 1.9 ^2^	−0.027 ± 0.000	0.980	<0.001
Poultry based dishes	27	0.6 ± 2.3	33	0.6 ± 2.1	20	1.5 ± 4.6 ^2,3^	0.031 ± 0.001	0.878	<0.001

^1^ *p*_trend_ from linear regression test for trends in means of the three surveys. ^2^ *p* < 0.001 compared with 1995NS. ^3^ *p* < 0.001 compared with 2007NS. ^4^ *β* ± SE indicates the change in unit of the food item per year.

**Table 4:** Per consumer mean ± SD comparison of the highest contributors to glycemic load in the three surveys.

Food Groups	1995NS			2007NS			2012NS			*β* ± SE ^5^	*R*^2^	*p*_trend_^2^
	Rank	Mean ± SD	% ^1^	Rank	Mean ± SD	% ^1^	Rank	Mean ± SD	% ^1^			
Flours, cereals and starches	1	22.8 ± 15.6	13.8	1	22.2 ± 15.4	18.7	1	27.3 ± 16.8	16.9	0.085 ± 0.009	0.061	<0.001
Bread and bread rolls	2	21.4 ± 12.1	84.5	2	20.2 ± 11.8 ^3^	77.3	3	20.3 ± 10.9	73.9	−0.071 ± 0.000	0.797	<0.001
Cereal based dishes	3	16.6 ± 11.9	23.6	6	15.2 ± 10.4	22.6	2	23.7 ± 14.3 ^3,4^	42.3	0.350 ± 0.010	0.348	<0.001
Breakfast cereals (hot porridge)	4	16.4 ± 7.7	2.5	3	17.5 ± 12.8	3.3	7	15.5 ± 11.3	5.3	−0.068 ± 0.006	0.295	<0.001
Breakfast cereals (ready to eat)	5	15.8 ± 9.2	58.0	4	17.2 ± 9.9 ^3^	53.4	8	15.3 ± 8.9 ^4^	43.9	0.067 ± 0.002	0.246	<0.001
Sweetened beverages	6	15.4 ± 11.5	38.2	11	12.2 ± 9.0 ^3^	31.1	16	10.5 ± 10.4 ^3,4^	37.2	−0.235 ± 0.001	0.945	<0.001
Potatoes	7	14.8 ± 10.5	50.7	9	13.3 ± 9.4 ^3^	42.9	12	12.5 ± 10.4 ^3^	34.3	−0.158 ± 0.001	0.893	<0.001
Batter-based products	8	14.4 ± 9.5	7.1	10	13.1 ± 10.1	12.6	11	12.6 ± 7.8	7.7	−0.125 ± 0.000	0.988	<0.001
Pastas	9	14.1 ± 8.9	15.4	5	15.7 ± 9.7	26.6	6	16.9 ± 9.9	10.9	0.097 ± 0.000	0.991	<0.001
Cake-type dessert	10	14.0 ± 10.4	22.5	8	14.1 ± 9.6	21.7	4	18.5 ± 12.4 ^3,4^	20.5	0.148 ± 0.006	0.245	<0.001
Fruits and vegetables juices	11	13.9 ± 10.0	71.2	14	10.9 ± 8.2 ^3^	59.3	14	11.2 ± 7.5 ^3^	39.4	−0.187 ± 0.001	0.896	<0.001
Fancy breads	12	13.0 ± 7.5	9.9	7	14.8 ± 9.4	18.0	5	17.5 ± 10.0 ^3,4^	16.6	0.193 ± 0.005	0.560	<0.001
Pastries	13	11.0 ± 8.1	17.8	13	11.9 ± 8.4	16.6	10	12.9 ± 8.7	14.4	0.153 ± 0.001	0.918	<0.001
Extruded snacks	14	9.8 ± 7.0	8.6	20	8.7 ± 6.5	2.5	15	10.8 ± 9.3	5.3	−0.029 ± 0.003	0.183	<0.001
Dried fruit, preserved fruit	15	8.7 ± 8.2	5.0	18	8.7 ± 7.8	6.9	19	9.2 ± 8.1	5.1	0.022 ± 0.001	0.518	<0.001
Other confectionery	16	8.6 ± 9.4	21.7	12	11.9 ± 11.6 ^3^	21.7	32	6.8 ± 6.4 ^4^	18.6	0.127 ± 0.006	0.222	<0.001
Sweet biscuits	17	8.2 ± 6.1	34.9	22	8.6 ± 6.5	31.6	18	9.5 ± 7.2 ^3^	35.5	0.052 ± 0.001	0.628	<0.001
Confectionery based dishes	18	8.0 ± 6.6	12.9	24	8.2 ± 6.4	9.3	24	7.7 ± 4.9	12.2	0.044 ± 0.001	0.511	<0.001
Savory biscuits	19	7.6 ± 6.0	20.2	16	9.9 ± 7.5 ^3^	27.1	13	12.1 ± 9.5 ^3,4^	26.7	0.196 ± 0.001	0.914	<0.001
Frozen milk products	20	7.4 ± 5.2	32.4	34	6.4 ± 4.8 ^3^	24.9	29	6.9 ± 4.7	22.7	−0.039 ± 0.001	0.497	<0.001
Tropical and subtropical fruit	21	7.2 ± 4.9	19.5	25	8.1 ± 5.1	25.1	20	9.2 ± 5.6 ^3^	21.6	0.088 ± 0.000	1.000	<0.001
Milk and milk products based dishes	22	7.0 ± 4.7	11.7	26	8.0 ± 4.8	6.4	31	6.9 ± 5.4	5.1	0.057 ± 0.004	0.220	<0.001
Fruit dishes	23	6.9 ± 3.7	0.5	15	10.9 ± 7.1	0.4	33	6.4 ± 7.5	0.7	−0.071 ± 0.057	0.038	0.219
Fruit combinations	24	6.6 ± 6.7	2.4	31	7.6 ± 4.4	3.1	23	8.6 ± 6.3	3.1	0.142 ± 0.000	0.998	<0.001
Flavored milks	25	6.6 ± 4.3	6.3	30	7.7 ± 4.3	8.2	9	13.1 ± 9.1 ^3,4^	9.6	0.234 ± 0.007	0.607	<0.001
Infant foods	26	6.4 ± 6.0	0.6	36	6.2 ± 4.8	0.4	#	9.3 ± 7.7	0.2	0.027 ± 0.007	0.326	<0.001
Chocolates	27	6.4 ± 6.2	26.7	23	8.3 ± 8.2	25.3	25	7.5 ± 7.8	19.1	0.061 ± 0.000	0.906	<0.001
Potato snacks	28	6.1 ± 4.1	18.4	33	6.9 ± 6.0	16.7	26	7.4 ± 7.2	17.5	0.022 ± 0.000	0.944	<0.001
Pretzels and other snacks	29	6.0 ± 3.5	0.7	35	6.4 ± 5.8	5.4	39	5.8 ± 5.0	4.6	−0.108 ± 0.001	0.980	<0.001
Cereal-, fruit-, nut-, seed-bars	30	6.0 ± 3.5	16.4	17	9.4 ± 5.6 ^3^	14.8	17	10.3 ± 5.2 ^3^	17.4	0.236 ± 0.000	0.998	<0.001
Infant formulae/breast milk	#	6.7 ± 15.8	0.04	21	8.7 ± 9.0	0.6	#	13.7 ± 17.3	0.4	−0.042 ± 0.048	0.023	0.388

Post-hoc analysis was not performed for food group with any group having less than 2 consumers (infant foods and infant formulae/breast milk). Groups with less than 10 consumers were excluded from the ranking (marked with #). ^1^ Percentage of participants who consumed foods in the food group; ^2^ *p*_trend_ from linear regression test for trends in medians of the three surveys; ^3^ *p* < 0.001 compared with 1995NS; ^4^ *p* < 0.001 compared with 2007NS; ^5^ *β* ± SE indicates the change in unit of the food item per year.

**Table 5:** The contribution of the top 20 GL food groups to inter-individual variations in dietary glycaemic index (dGI) and glycaemic load (dGL) in 1995NS (*n* = 2475).

Food Groups	dGI			dGL		
	*β* ± SE	*Partial R*^2^	*P* Value	*β* ± SE	*Partial R*^2^	*p* Value
	Model *R*^2^ = 0.441			Model *R*^2^ = 0.888		
Bread and bread rolls	2.14 ± 0.14	0.087	<0.001	17.20 ± 0.72	0.188	<0.001
Fruits and vegetables juices	−0.13 ± 0.02	0.013	<0.001	3.11 ± 0.12	0.208	<0.001
Breakfast cereals (ready to eat)	3.15 ± 0.23	0.070	<0.001	33.25 ± 1.20	0.237	<0.001
Potatoes	1.64 ± 0.08	0.133	<0.001	6.56 ± 0.44	0.084	<0.001
Sweetened beverages	0.26 ± 0.03	0.031	<0.001	4.18 ± 0.15	0.231	<0.001
Cereal based dishes	0.35 ± 0.07	0.009	<0.001	4.15 ± 0.37	0.049	<0.001
Dairy milk	−0.43 ± 0.03	0.077	<0.001	−0.91 ± 0.16	0.014	<0.001
Cake-type dessert	−0.34 ± 0.16	0.002	0.034	10.33 ± 0.83	0.060	<0.001
Flours, cereals and starches	1.12 ± 0.09	0.056	<0.001	10.60 ± 0.48	0.164	<0.001
Sweet biscuits	0.28 ± 0.35	<0.001	0.421	10.19 ± 1.80	0.013	<0.001
Frozen milk products	−0.68 ± 0.10	0.019	<0.001	2.07 ± 0.51	0.007	<0.001
Pastas	−0.93 ± 0.10	0.038	<0.001	2.68 ± 0.49	0.012	<0.001
Sugar, honey and syrups	1.46 ± 0.49	0.004	0.003	27.34 ± 2.54	0.045	<0.001
Pome fruit	−0.92 ± 0.07	0.059	<0.001	1.55 ± 0.39	0.007	<0.001
Pastries	−0.43 ± 0.11	0.007	<0.001	0.22 ± 0.56	<0.001	0.697
Other confectionery	4.37 ± 0.40	0.046	<0.001	46.43 ± 2.09	0.167	<0.001
Chocolates	−1.33 ± 0.33	0.007	<0.001	7.90 ± 1.70	0.009	<0.001
Savory biscuits	2.22 ± 0.49	0.008	<0.001	14.20 ± 2.53	0.013	<0.001
Tropical and subtropical fruit	−0.68 ± 0.16	0.008	<0.001	4.55 ± 0.81	0.013	<0.001
Fancy breads	1.48 ± 0.32	0.009	<0.001	12.88 ± 1.65	0.024	<0.001

*β* ± SE calculated using multiple linear regression, with energy and the food groups as the predictor variables. *β* expressed as change in dGI or dGL per 100 g increase in intake of the corresponding food group.

**Table 6:** The contribution of the top 20 GL food groups to inter-individual variations in dietary glycaemic index (dGI) and glycaemic load (dGL) in 2007NS (*n* = 4373).

Food Groups	dGI			dGL		
	Food Groups	*β* ± SE	*Partial R*^2^	*p* Value	*β* ± SE	*Partial R*^2^	*p* Value
	Model *R*^2^ = 0.372			Model *R*^2^ = 0.862		
Breads, and bread rolls	2.62 ± 0.13	0.091	<0.001	15.45 ± 0.57	0.145	<0.001
Breakfast cereals (ready to eat)	3.93 ± 0.19	0.086	<0.001	33.54 ± 0.88	0.250	<0.001
Fruit and vegetables juices	0.05 ± 0.03	0.001	0.118	3.08 ± 0.15	0.094	<0.001
Potatoes	1.83 ± 0.08	0.102	<0.001	7.49 ± 0.37	0.085	<0.001
Pastas	−0.54 ± 0.07	0.015	<0.001	4.92 ± 0.30	0.059	<0.001
Flours, cereals and starches	1.71 ± 0.08	0.097	<0.001	13.60 ± 0.36	0.250	<0.001
Sweetened beverages	0.23 ± 0.03	0.017	<0.001	2.89 ± 0.12	0.114	<0.001
Dairy milk	−0.36 ± 0.03	0.035	<0.001	−0.58 ± 0.13	0.004	<0.001
Cereal based dishes	0.01 ± 0.07	<0.001	0.850	3.64 ± 0.30	0.033	<0.001
Cake-type dessert	0.02 ± 0.16	<0.001	0.900	8.16 ± 0.74	0.028	<0.001
Fancy breads	1.40 ± 0.21	0.010	<0.001	12.46 ± 0.94	0.039	<0.001
Savory biscuits	3.89 ± 0.39	0.022	<0.001	19.49 ± 1.78	0.027	<0.001
Sweet biscuits	1.52 ± 0.32	0.005	<0.001	16.71 ± 1.46	0.029	<0.001
Other confectionery	5.68 ± 0.28	0.086	<0.001	48.19 ± 1.28	0.247	<0.001
Pome fruit	−0.83 ± 0.07	0.036	<0.001	2.19 ± 0.30	0.012	<0.001
Chocolates	−0.15 ± 0.28	<0.001	0.595	10.68 ± 1.26	0.016	<0.001
Pastries	0.10 ± 0.10	<0.001	0.324	2.32 ± 0.45	0.006	<0.001
Tropical and subtropical fruit	−0.42 ± 0.10	0.004	<0.001	3.40 ± 0.47	0.012	<0.001
Sugar, honey and syrups	2.01 ± 0.48	0.004	<0.001	29.61 ± 2.16	0.041	<0.001
Batter-based product	2.66 ± 0.22	0.032	<0.001	16.03 ± 1.01	0.055	<0.001

*β* ± SE calculated using multiple linear regression, with energy and the food groups as the predictor variables. *β* expressed as change in dGI or dGL per 100 g increase in intake of the corresponding food group.

**Table 7:** The contribution of the top 20 GL food groups to inter-individual variations in dietary glycaemic index (dGI) and glycaemic load (dGL) in 2012NS (*n* = 1691).

Food Groups	dGI			dL		
	Food Groups	*β* ± SE	*Partial R*^2^	*p* Value	*β* ± SE	*Partial R*^2^	*p* Value
	Model *R*^2^ = 0.351			Model *R*^2^ = 0.846		
Breads, and bread rolls	1.94 ± 0.21	0.049	<0.001	13.18 ± 0.97	0.101	<0.001
Cereal based dishes	0.53 ± 0.07	0.034	<0.001	3.83 ± 0.32	0.077	<0.001
Breakfast cereals (ready to eat)	2.93 ± 0.37	0.036	<0.001	24.95 ± 1.72	0.112	<0.001
Fruit and vegetables juices	−0.12 ± 0.05	0.003	0.030	2.82 ± 0.25	0.069	<0.001
Flours, cereals and starches	2.06 ± 0.14	0.119	<0.001	16.05 ± 0.63	0.278	<0.001
Potatoes	1.54 ± 0.13	0.076	<0.001	6.52 ± 0.61	0.064	<0.001
Sweetened beverages	0.13 ± 0.04	0.008	<0.001	3.22 ± 0.17	0.185	<0.001
Cake-type dessert	−0.79 ± 0.20	0.010	<0.001	6.24 ± 0.90	0.028	<0.001
Sweet biscuits	−0.18 ± 0.42	<0.001	0.660	13.67 ± 1.94	0.029	<0.001
Savory biscuits	3.17 ± 0.43	0.032	<0.001	18.26 ± 1.98	0.048	<0.001
Dairy milk	−0.56 ± 0.05	0.077	<0.001	−1.14 ± 0.22	0.016	<0.001
Fancy breads	1.13 ± 0.30	0.008	<0.001	10.56 ± 1.40	0.033	<0.001
Pome fruit	−0.81 ± 0.09	0.045	<0.001	1.40 ± 0.42	0.007	0.001
Tropical and subtropical fruit	−0.68 ± 0.18	0.009	<0.001	3.44 ± 0.83	0.010	<0.001
Pastries	−0.64 ± 0.16	0.009	<0.001	0.21 ± 0.76	<0.001	0.786
Pastas	0.07 ± 0.14	<0.001	0.601	3.16 ± 0.63	0.015	<0.001
Cereal-, fruit-, nut-, and seed-bars	3.14 ± 0.73	0.011	<0.001	21.13 ± 3.36	0.023	<0.001
Sugar, honey and syrups	4.27 ± 0.82	0.016	<0.001	43.35 ± 3.78	0.073	<0.001
Frozen milk products	−1.03 ± 0.19	0.017	<0.001	−0.41 ± 0.88	<0.001	0.640
Poultry based dishes	0.44 ± 0.15	0.005	0.003	0.17 ± 0.68	<0.001	0.804

*β* ± SE calculated using multiple linear regression, with energy and the food groups as the predictor variables. *β* expressed as change in dGI or dGL per 100 g increase in intake of the corresponding food group.

## Appendix A

**Table A1:** Re-coding of food groups in the AUSNUT databases.

New Code	New Food Group	1995NS Code	1995NS Food Group	2007NS Code	2007NS Food Group	2012NS Code	2012NS Food Group
111	Tea	111	Tea	111	Tea	111	Tea
112	Coffee and coffee substitute	112	Coffee and coffee substitutes	112	Coffee and coffee substitutes	112	Coffee and coffee substitutes
113	Fruit and vegetable juices and drinks	113	Fruit and vegetables juices and drinks	113	Fruit and vegetable juices, and drinks	113	Fruit and vegetables juices and drinks
				114	Cordials	114	Cordials
114	Sweetened beverages	114	Soft drinks, flavored mineral waters and electrolyte drinks	115	Soft drinks, and flavored mineral waters	115	Soft drinks, and flavored mineral waters
				116	Electrolyte, energy and fortified drinks	116	Electrolyte, energy and fortified drinks
115	Mineral waters	115	Mineral waters and water	117	Mineral waters and water	117	Waters, municipal and bottled, unflavored
116	Other beverage flavorings and prepared beverages	116	Water with other additions as a beverage	118	Other beverage flavorings and prepared beverages	118	Other beverage flavorings and prepared beverages
		301	Beverage flavorings				
121	Flours and other cereal grains and starches	121	Flours and other cereal grains and starches	121	Flours and other cereal grains and starches	121	Flours and other cereal grains and starches
		126	Rice and rice products				
122	Bread and bread rolls	122	Regular breads, and rolls	122	Regular breads, and bread rolls (plain/unfilled/untopped varieties)	122	Regular breads, and bread rolls (plain/unfilled/untopped varieties)
123	Breakfast cereals (ready to eat)	123	BF cereals, plain, single source	125	Breakfast cereals and bars, unfortified and fortified varieties	125	Breakfast cereals, ready to eat
124	Fancy breads	124	Fancy breads, flat breads, English-style muffins and crumpets	123	English-style muffins, flat breads, and savoury and sweet breads	123	English-style muffins, flat breads, and savory and sweet breads
125	Pasta and pasta products	125	Pasta and pasta products	124	Pasta and pasta products	124	Pasta and pasta products (without sauce)
127	Breakfast Cereals, Mixed Source						
128	Breakfast cereals (hot porridge)	128	Bf cereal, hot porridge type	126	Breakfast cereal, hot porridge type	126	Breakfast cereals, hot porridge style
131	Sweet biscuits	131	Sweet biscuit	131	Sweet biscuits	131	Sweet biscuits
132	Savory biscuits	132	Savory biscuit	132	Savory biscuits	132	Savory biscuits
133	Cake-type desserts	133	Cakes, buns, muffins, scones, cake-type desserts	133	Cakes, buns, muffins, scones, cake-type desserts	133	Cakes, muffins, scones, cake-type desserts
134	Pastries	134	Pastries	134	Pastries	134	Pastries
135	Cereal-based dishes	135	Mixed dishes where cereal is the major ingredient	135	Mixed dishes where cereal is the major ingredient	135	Mixed dishes where cereal is the major ingredient
136	Batter-based products	136	Batter-based products	136	Batter-based products	136	Batter-based products
141	Butters	141	Dairy fats	141	Butters	141	Butters
				142	Dairy blends	142	Dairy blends
142	Margarine and table spreads	142	Margarine	143	Margarine and table spreads	143	Margarine and table spreads
143	Vegetable oils	143	Vegetable oil	144	Vegetable/nut oil	144	Plant oils
144	Other fats	144	Other fats	145	Other fats	145	Other fats
145	Unspecified fats	145	Unspecified fats	146	Unspecified fats	146	Unspecified fats
151	Fin fish (excluding canned)	151	Fin fish (excluding canned)	151	Fin fish (excluding commercially sterile)	151	Fin fish (excluding commercially sterile)
152	Crustacea and molluscs (excluding canned)	152	Crustacea and molluscs (excluding canned)	152	Crustacea and molluscs (excluding commercially sterile)	152	Crustacea and molluscs (excluding commercially sterile)
153	Other sea and freshwater foods	153	Other sea and freshwater foods	153	Other sea and freshwater foods	153	Other sea and freshwater foods
154	Packed (canned and bottled) fish and seafood	154	Packed (canned and bottled) fish and seafood	154	Packed (commercially sterile) fish and seafood	154	Packed (commercially sterile) fish and seafood
155	Fish and seafood products	155	Fish and seafood products	155	Fish and seafood products (homemade and takeaway)	155	Fish and seafood products (homemade and takeaway)
156	Mixed dishes with fish or seafood as the major component	156	Mixed dishes with fish or seafood as the major component	156	Mixed dishes with fish or seafood as the major component	156	Mixed dishes with fish or seafood as the major component
161	Pome fruit	161	Pome fruit	161	Pome fruit	161	Pome fruit
162	Berry fruit	162	Berry fruit	162	Berry fruit	162	Berry fruit
163	Citrus fruit	163	Citrus fruit	163	Citrus fruit	163	Citrus fruit
164	Stone fruit	164	Stone fruit	164	Stone fruit	164	Stone fruit
165	Tropical fruit	165	Tropical fruit	165	Tropical fruit	165	Tropical and subtropical fruit
166	Other fruit	166	Other fruit	166	Other fruit	166	Other fruit
167	Fruit combinations	167	Mixtures of two or more groups of fruit	167	Mixtures of two or more groups of fruit	167	Mixtures of two or more groups of fruit
168	Dried fruits	168	Dried fruit, preserved fruit	168	Dried fruit, preserved fruit	168	Dried fruit, preserved fruit
169	Fruit dishes	169	Mixed dishes where fruit is the major component	169	Mixed dishes where fruit is the major component	169	Mixed dishes where fruit is the major component
171	Eggs	171	Eggs	171	Eggs	171	Eggs
				173	Egg substitutes and dishes		
172	Egg-based dishes	172	Dishes where egg is the major ingredient	172	Dishes where egg is the major ingredient	172	Dishes where egg is the major ingredient
181	Muscle meat	181	Muscle meat	181	Muscle meat	181	Beef, sheep and pork, unprocessed
182	Game and other carcase meats	182	Game and other carcase meats	182	Game and other carcase meats	182	Mammalian game meats
183	Poultry and feathered game	183	Poultry and feathered game	183	Poultry and feathered game	183	Poultry and feathered game
184	Organ meats and offal, products and dishes	184	Organ meats and offal, products and dishes	184	Organ meats and offal, products and dishes	184	Organ meats and offal, products and dishes
185	Sausages, frankfurts and saveloys	185	Sausages, frankfurts and saveloys	185	Sausages, frankfurts and saveloys	185	Sausages, frankfurts and saveloys
186	Processed meat	186	Processed meat	186	Processed meat	186	Processed meat
187	Mixed dishes where beef or veal is the major component	187	Mixed dishes where beef or veal is the major component	187	Mixed dishes where beef, veal or lamb is the major component	187	Mixed dishes where beef, sheep, pork or mammalian game is the major component
188	Mixed dishes where lamb or pork, bacon, ham is the major component	188	Mixed dishes where lamb or pork, bacon, ham is the major component	188	Mixed dishes where pork, bacon, ham is the major component	188	Mixed dishes where sausage, bacon, ham or other processed meat is the major component
189	Mixed dishes where poultry or game is the major component	189	Mixed dishes where poultry or game is the major component	189	Mixed dishes where poultry or game is the major component	189	Mixed dishes where poultry or feathered game is the major component
191	Dairy milk	191	Dairy milk	191	Dairy milk (cow, sheep and goat)	191	Dairy milk (cow, sheep and goat)
192	Yogurt	192	Yogurt	192	Yoghurt	192	Yoghurt
193	Cream	193	Cream	193	Cream	193	Cream
194	Cheese	194	Cheese	194	Cheese	194	Cheese
195	Frozen milk products	195	Frozen milk products	195	Frozen milk products	195	Frozen milk products
196	Milk and milk products based dishes	196	Other dishes where milk or a milk product is the major component	196	Custards	196	Custards
				197	Other dishes where milk or a milk product is the major component	197	Other dishes where milk or a milk product is the major component
197	Milk substitutes	197	Milk substitutes	201	Dairy milk substitutes, unflavored	201	Dairy milk substitutes, unflavored
				202	Dairy milk substitutes, flavored	202	Dairy milk substitutes, flavored
				203	Cheese substitute	203	Cheese substitute
				204	Soy-based ice confection	204	Soy-based ice confection
				205	Soy-based yoghurts	205	Soy-based yoghurts
198	Flavored milks	198	Flavored milks	198	Flavored milks	198	Flavored milks and milkshakes
201	Soup	201	Soup	211	Soup (prepared, ready to eat)	211	Soup, homemade from basic ingredients
						213	Soup, prepared from dry soup mix
202	Dry soup mix	202	Dry soup mix	212	Dry soup mix	212	Dry soup mix
203	Canned condensed soup	203	Canned condensed soup	213	Canned condensed soup (unprepared)	214	Canned condensed soup (unprepared)
						215	Soup, commercially sterile, prepared from condensed or sold ready to eat
						216	Soup, not commercially sterile, purchased ready to eat
211	Seeds and seed products	211	Seeds and seed products	221	Seeds and seed products	221	Seeds and seed products
212	Nuts and nuts products	212	Nuts and nuts products	222	Nuts and nut products	222	Nuts and nut products
221	Gravies and savoury sauces	221	Gravies and savoury sauces	231	Gravies and savoury sauces	231	Gravies and savoury sauces
						235	Dips
222	Pickles, chutneys and relishes	222	Pickles, chutneys and relishes	232	Pickles, chutneys and relishes	232	Pickles, chutneys and relishes
224	Salad dressings	224	Salad dressings	233	Salad dressings	233	Salad dressings
225	Stuffings	225	Stuffings	234	Stuffings	234	Stuffings
231	Potatoes	231	Potatoes	241	Potatoes	241	Potatoes
232	Cabbage, cauliflower and similar brassica vegetables	232	Cabbage, cauliflower and similar brassica vegetables	242	Cabbage, cauliflower and similar brassica vegetables	242	Cabbage, cauliflower and similar brassica vegetables
233	Carrot and similar root vegetables	233	Carrot and similar root vegetables	243	Carrot and similar root vegetables	243	Carrot and similar root vegetables
234	Leaf and stalk vegetables	234	Leaf and stalk vegetables	244	Leaf and stalk vegetables	244	Leaf and stalk vegetables
235	Peas and beans	235	Peas and beans	245	Peas and beans	245	Peas and beans
236	Tomato and tomato products	236	Tomato and tomato products	246	Tomato and tomato products	246	Tomato and tomato products
237	Other fruiting vegetables	237	Other fruiting vegetables	247	Other fruiting vegetables	247	Other fruiting vegetables
238	Other vegetables and vegetable combinations	238	Other vegetables and vegetable combinations	248	Other vegetables and vegetable combinations	248	Other vegetables and vegetable combinations
239	Dishes where vegetable is the major component	239	Dishes where vegetable is the major component	249	Dishes where vegetable is the major component	249	Dishes where vegetable is the major component
241	Mature legumes and pulses	241	Mature legumes and pulses	251	Mature legumes and pulses	251	Mature legumes and pulses
242	Mature legume and pulse products and dishes	242	Mature legume and pulse products and dishes	252	Mature legume and pulse products and dishes	206	Meat substitutes
						207	Dishes where meat substitutes are the major component
						252	Mature legume and pulse products and dishes
251	Potato snacks	251	Potato snacks	261	Potato snacks	261	Potato snacks
252	Corn snacks	252	Corn snacks	262	Corn snacks	262	Corn snacks
253	Extruded snacks	253	Extruded snacks	263	Extruded or reformed snacks	263	Extruded or reformed snacks
254	Pretzels and other snacks	254	Pretzels and other snacks	264	Pretzels	264	Other snacks
				265	Other snacks		
261	Sugar, honey and syrups	261	Sugar, honey and syrups	271	Sugar, honey and syrups	271	Sugar, honey and syrups
262	Jam and lemon spreads, chocolate spreads	262	Jam and lemon spreads, chocolate spreads	272	Jam and lemon spreads, chocolate spreads, sauces	272	Jam and lemon spreads, chocolate spreads, sauces
263	Dishes and products other than confectionery where sugar is the major component	263	Dishes and products other than confectionery where sugar is the major component	273	Dishes & products other than confectionery where sugar is major component	273	Dishes and products other than confectionery where sugar is the major component
271	Chocolate and chocolate-based confectionery	271	Chocolate and chocolate-based confectionery	281	Chocolate and chocolate-based confectionery	281	Chocolate and chocolate-based confectionery
272	Cereal-, fruit-, nut-, and seed-bars	272	Cereal-, fruit-, nut-, and seed-bars	282	Cereal-, fruit-, nut- and seed-bars	282	Fruit, nut and seed-bars
						283	Muesli or cereal style bars
273	Other confectionery	273	Other confectionery	283	Other confectionery	284	Other confectionery
281	Beers	281	Beers	291	Beers	291	Beers
282	Wines	282	Wines	292	Wines	292	Wines
283	Spirits	283	Spirits	293	Spirits	293	Spirits
284	Other alcoholic beverages	284	Other alcoholic beverages	294	Other alcoholic beverages	294	Cider and perry
				295	Pre-mixed drinks	295	Other alcoholic beverages
291	Formula dietary foods	291	Formula dietary foods	301	Formula dietary foods	301	Formula dietary foods
		292	Enteral formula	302	Enteral formula		
302	Yeast: yeast, vegetable and meat extracts	302	Yeast: yeast, vegetable and meat extracts	311	Yeast, yeast, vegetable and meat extracts	311	Yeast, and yeast vegetable or meat extracts
303	Artificial sweetening agents	303	Artificial sweetening agents	312	Intense sweetening agents	312	Intense sweetening agents
304	Herbs, spices, seasonings and stock cubes	304	Herbs, spices, seasonings and stock cubes	313	Herbs, spices, seasonings and stock cubes	313	Herbs, spices, seasonings and stock cubes
		305	Essences	314	Essences	314	Essences
306	Chemical raising agents and cooking ingredients	306	Chemical raising agents and cooking ingredients	315	Chemical raising agents and cooking ingredients	315	Chemical raising agents and cooking ingredients
311	Infant formulae and human breast milk	311	Infant formulae and human breast milk	321	Infant formulae and human breast milk	321	Infant formulae and human breast milk
312	Infant cereal products	312	Infant cereal products	322	Infant cereal products	322	Infant cereal products
313	Infant foods	313	Infant foods	323	Infant foods	323	Infant foods
314	Infant drinks	314	Infant drinks	324	Infant drinks	324	Infant drinks

**Table A2:** *Per capita* mean ± SD comparison of the highest contributors to glycemic load in the three surveys—all subjects included.

Food Groups	1995NS		2007NS		2012NS		*β* ± SE	*R*^2^	*p*_trend_^1^
	Rank	Mean ± SD	Rank	Mean ± SD	Rank	Mean ± SD			
Bread and bread rolls	1	18.3 ± 14.0	1	15.6 ± 13.6 ^2^	1	15.4 ± 13.9 ^2^	−0.199 ± 0.000	0.964	<0.001
Fruit and vegetable juices	2	10.0 ± 10.8	3	6.5 ± 8.5 ^2^	4	5.8 ± 8.6 ^2^	−0.277 ± 0.000	0.992	<0.001
Breakfast cereals (ready to eat)	3	9.1 ± 10.7	2	9.1 ± 11.4	3	7.0 ± 10.0 ^2,3^	−0.084 ± 0.001	0.332	<0.001
Potatoes	4	7.5 ± 10.7	4	5.7 ± 9.1 ^2^	6	4.3 ± 8.7 ^2,3^	−0.181 ± 0.000	0.950	<0.001
Sweetened beverages	5	5.9 ± 10.4	7	3.9 ± 7.7 ^2^	7	4.0 ± 9.0 ^2^	−0.132 ± 0.000	0.890	<0.001
Cereal based dishes	6	3.9 ± 9.1	9	3.6 ± 8.6	2	9.8 ± 15.0 ^2,3^	0.229 ± 0.004	0.287	<0.001
Cake-type dessert	7	3.2 ± 7.8	10	3.0 ± 7.3	8	3.9 ± 9.5 ^3^	0.023 ± 0.001	0.167	<0.001
Flours, cereals and starches	8	3.1 ± 9.8	6	4.1 ± 11.0 ^2^	5	4.6 ± 12.2 ^2^	0.088 ± 0.000	0.995	<0.001
Dairy milk	9	3.1 ± 3.6	8	3.8 ± 4.2 ^2^	10	3.1 ± 4.2 ^3^	0.019 ± 0.001	0.126	<0.001
Sweet biscuits	10	2.8 ± 5.3	12	2.7 ± 5.6	9	3.2 ± 6.4 ^3^	0.015 ± 0.000	0.161	<0.001
Frozen milk products	11	2.4 ± 4.7	21	1.6 ± 3.7 ^2^	20	1.5 ± 3.6 ^2^	−0.060 ± 0.000	0.972	<0.001
Pastas	12	2.2 ± 6.2	5	4.2 ± 8.7 ^2^	17	1.8 ± 6.3 ^3^	0.044 ± 0.002	0.059	<0.001
Sugar, honey and syrups	13	2.1 ± 3.8	19	1.8 ± 3.8	18	1.6 ± 3.8 ^2^	−0.027 ± 0.000	0.911	<0.001
Pome fruit	14	2.0 ± 3.6	15	2.3 ± 3.9	12	2.7 ± 4.3 ^2,3^	0.035 ± 0.000	0.810	<0.001
Pastries	15	1.9 ± 5.3	17	2.0 ± 5.7	16	1.8 ± 5.5	−0.001 ± 0.000	0.006	<0.001
Other confectionery	16	1.9 ± 5.7	14	2.5 ± 7.3 ^2^	22	1.2 ± 3.9 ^3^	−0.005 ± 0.001	0.004	<0.001
Chocolates	17	1.7 ± 4.2	16	2.1 ± 5.5	21	1.5 ± 4.5 ^3^	0.002 ± 0.000	0.002	<0.001
Savory biscuits	18	1.5 ± 4.2	13	2.6 ± 5.9 ^2^	11	3.0 ± 7.2 ^2^	0.093 ± 0.000	0.999	<0.001
Tropical and subtropical fruit	19	1.4 ± 3.6	18	1.9 ± 4.3 ^2^	14	1.9 ± 4.5 ^2^	0.038 ± 0.000	0.925	<0.001
Fancy breads	20	1.3 ± 4.7	11	2.8 ± 7.3 ^2^	13	2.6 ± 7.5 ^2^	0.095 ± 0.000	0.871	<0.001
Potato snacks	21	1.1 ± 3.0	25	1.2 ± 3.6	23	1.2 ± 3.9	0.006 ± 0.000	0.929	<0.001
Batter-based product	22	1.0 ± 4.5	20	1.7 ± 5.7 ^2^	28	0.9 ± 4.0 ^3^	0.015 ± 0.001	0.068	<0.001
Confectionery based dishes	23	1.0 ± 3.5	28	0.8 ± 3.1	26	1.0 ± 3.4	−0.007 ± 0.000	0.162	<0.001
Cereal-, fruit-, nut-,seed-bars	24	1.0 ± 2.6	22	1.3 ± 3.9 ^2^	15	1.9 ± 4.7 ^2,3^	0.048 ± 0.000	0.804	<0.001
Extruded snacks	25	0.8 ± 3.5	43	0.2 ± 1.7 ^2^	34	0.5 ± 3.0 ^2,3^	−0.031 ± 0.000	0.538	<0.001
Milk and milk products based dishes	26	0.8 ± 2.7	36	0.5 ± 2.3 ^2^	39	0.4 ± 2.1 ^2^	−0.025 ± 0.000	0.994	<0.001
Gravies and savory sauces	27	0.6 ± 1.3	27	0.8 ± 1.8 ^2^	33	0.6 ± 2.0 ^3^	0.006 ± 0.000	0.098	<0.001
Poultry based dishes	28	0.6 ± 2.2	33	0.6 ± 2.2	19	1.5 ± 4.9 ^2,3^	0.038 ± 0.001	0.341	<0.001

^1^ *P*_trend_ from linear regression test for trends in means of the three surveys. ^2^ *p* < 0.001 compared with 1995NS. ^3^ *p* < 0.001 compared with 2007NS.

**Table A3:** Per consumer mean ± SD comparison of the highest contributors to glycemic load in the three surveys—all subjects included.

Food Groups	1995NS			2007NS			2012NS			*β* ± SE	*R*^2^	*P*_trend_^2^
	Rank	Mean ± SD	% ^1^	Rank	Mean ± SD	% ^1^	Rank	Mean ± SD	% ^1^			
Flour, cereals and starches	1	23.0 ± 15.7	13.7	1	22.6 ± 15.8	18.3	1	27.1 ± 16.6 ^4^	16.9	0.144 ± 0.009	0.138	<0.001
Bread and bread rolls	2	21.8 ± 12.6	84.2	2	20.5 ± 12.0 ^3^	76.4	3	21.2 ± 12.0	72.5	−0.038 ± 0.001	0.126	<0.001
Cereal-based dishes	3	16.5 ± 11.8	23.7	6	16.0 ± 11.4	22.6	2	24.1 ± 14.5 ^3,4^	40.8	0.431 ± 0.010	0.395	<0.001
Breakfast cereals (hot porridge)	4	16.1 ± 8.3	2.7	3	18.2 ± 13.0	3.3	8	15.3 ± 10.8	5.3	−0.050 ± 0.006	0.145	<0.001
Breakfast cereals (ready to eat)	5	16.0 ± 9.4	56.6	4	17.3 ± 10.2 ^3^	52.3	7	15.9 ± 9.3 ^4^	43.9	0.046 ± 0.001	0.193	<0.001
Sweetened beverages	6	15.5 ± 11.8	37.8	11	12.4 ± 9.3 ^3^	31.4	18	10.9 ± 12.0 ^3^	36.9	−0.258 ± 0.001	0.919	<0.001
Potatoes	7	14.9 ± 10.8	50.2	9	13.4 ± 9.5 ^3^	42.5	10	12.9 ± 10.6 ^3^	33.7	−0.157 ± 0.000	0.996	<0.001
Batter-based products	8	14.5 ± 9.7	7.0	10	13.2 ± 10.3	12.5	11	12.7 ± 8.6	7.2	−0.125 ± 0.000	0.986	<0.001
Cake-type desserts	9	14.3 ± 10.8	22.2	8	14.2 ± 9.8	21.1	4	19.1 ± 12.6 ^3,4^	20.2	0.204 ± 0.007	0.278	<0.001
Fruit and vegetable juices	10	14.2 ± 10.3	70.7	14	11.1 ± 8.5 ^3^	58.4	16	11.6 ± 8.1 ^3^	39.8	−0.180 ± 0.001	0.807	<0.001
Pastas	11	14.1 ± 9.2	15.3	5	16.0 ± 9.9	26.2	5	17.7 ± 10.6 ^3^	10.0	0.128 ± 0.000	0.998	<0.001
Fancy breads	12	13.1 ± 7.8	10.1	7	15.3 ± 10.1	18.0	6	17.6 ± 10.7 ^3,4^	14.8	0.204 ± 0.005	0.548	<0.001
Pastries	13	10.8 ± 8.1	17.6	12	12.1 ± 8.4	16.7	12	12.7 ± 8.9	14.0	0.168 ± 0.000	1.000	<0.001
Extruded snacks	14	9.9 ± 7.4	8.6	18	8.9 ± 6.4	2.5	17	11.1 ± 8.5	4.7	0.037 ± 0.005	0.101	<0.001
Other confectionery	15	8.8 ± 9.5	21.3	13	12.1 ± 11.8 ^3^	21.1	34	7.0 ± 6.7 ^4^	17.7	0.100 ± 0.005	0.162	<0.001
Dried fruit, preserved fruit	16	8.8 ± 8.3	4.8	19	8.8 ± 8.1	6.7	20	9.7 ± 8.1	4.9	0.041 ± 0.002	0.377	<0.001
Pretzels and other snacks	17	8.4 ± 10.8	0.7	36	6.3 ± 5.7	5.2	37	6.9 ± 6.7	4.9	−0.094 ± 0.002	0.813	<0.001
Sweet biscuit	18	8.2 ± 6.2	34.1	20	8.7 ± 7.0	30.5	21	9.6 ± 7.8 ^3^	33.7	0.061 ± 0.001	0.728	<0.001
Confectionery-based dishes	19	8.0 ± 6.7	12.4	23	8.4 ± 6.8	9.0	26	8.3 ± 6.3	11.7	0.064 ± 0.000	0.997	<0.001
Savory biscuit	20	7.6 ± 6.3	20.2	16	9.9 ± 7.6 ^3^	26.5	15	11.7 ± 9.8 ^3,4^	26.0	0.187 ± 0.001	0.953	<0.001
Frozen milk products	21	7.5 ± 5.5	31.8	34	6.5 ± 5.0 ^3^	24.3	36	6.9 ± 4.8	21.5	−0.043 ± 0.001	0.462	<0.001
Tropical and subtropical fruit	22	7.3 ± 5.0	19.1	27	8.1 ± 5.2	23.9	23	9.1 ± 5.4 ^3^	21.4	0.086 ± 0.000	0.998	<0.001
Milk and milk products based dishes	23	6.9 ± 4.7	11.5	29	8.1 ± 5.0	6.3	30	7.3 ± 5.7	5.2	0.056 ± 0.004	0.194	<0.001
Fruit dishes	24	6.9 ± 3.7	0.5	15	10.9 ± 7.1	0.4	35	6.9 ± 8.5	0.5	-0.069 ± 0.055	0.038	0.217
Infant formulae / breast milk	#	6.7 ± 15.8	0.0	24	8.4 ± 8.7	0.6	9	15.3 ± 15.6	0.5	0.113 ± 0.058	0.292	0.057
Chocolates	25	6.4 ± 6.1	26.5	22	8.4 ± 8.4 ^3^	24.7	27	7.7 ± 7.5	19.0	0.061 ± 0.000	0.891	<0.001
Fruit combinations	26	6.4 ± 6.4	2.5	28	8.1 ± 5.5	3.0	22	9.6 ± 7.3	3.3	0.200 ± 0.001	0.988	<0.001
Flavored milks	27	6.4 ± 4.2	6.5	32	7.6 ± 4.3	8.1	14	12.7 ± 9.0 ^3,4^	8.5	0.246 ± 0.007	0.649	<0.001
Potato snacks	28	6.1 ± 4.3	18.4	33	7.1 ± 6.1	16.7	32	7.1 ± 6.6	17.2	0.021 ± 0.000	0.935	<0.001
Infant foods	29	6.1 ± 5.8	0.6	35	6.4 ± 5.0	0.4	#	9.3 ± 7.2	0.2	0.081 ± 0.005	0.878	<0.001
Cereal-, fruit-, nut-, and seed-bars	30	6.0 ± 3.6	15.9	17	9.4 ± 5.7 ^3^	14.2	19	10.6 ± 5.5 ^3,4^	17.7	0.243 ± 0.000	1.000	<0.001
Fish and seafood products	31	5.8 ± 5.1	5.0	44	3.9 ± 3.0 ^3^	5.2	31	7.2 ± 5.5 ^4^	3.7	0.028 ± 0.007	0.028	<0.001
Other fruits	32	5.8 ± 6.1	9.9	30	7.9 ± 6.7 ^3^	16.1	38	6.3 ± 5.4 ^4^	15.2	0.083 ± 0.004	0.230	<0.001
Pome fruits	33	5.7 ± 4.0	34.8	37	6.0 ± 4.1	37.9	33	7.1 ± 4.2 ^3,4^	37.2	0.053 ± 0.001	0.390	<0.001
Soups	34	5.7 ± 5.9	6.1	25	8.3 ± 6.5 ^3^	6.6	42	5.3 ± 6.2 ^4^	5.0	0.064 ± 0.008	0.101	<0.001
Legume and legume-based dishes	35	5.5 ± 6.0	4.2	42	4.6 ± 4.5	4.7	40	6.1 ± 6.6	3.3	-0.026 ± 0.003	0.201	<0.001
Vegetable-based dishes	36	5.5 ± 5.7	1.6	49	2.8 ± 6.1	7.0	56	1.5 ± 4.7 ^3^	13.4	-0.251 ± 0.004	0.854	<0.001
Corn snacks	37	5.3 ± 5.7	6.5	21	8.6 ± 7.6 ^3^	7.5	28	7.3 ± 7.8	6.8	0.102 ± 0.003	0.568	<0.001
Yogurt	38	4.7 ± 4.3	8.1	41	4.8 ± 3.9	19.6	47	4.1 ± 5.1	18.2	-0.048 ± 0.002	0.257	<0.001
Sugar, honey and syrups	39	4.7 ± 4.5	45.1	39	5.1 ± 4.8	36.0	39	6.2 ± 5.3 ^3,4^	26.3	0.058 ± 0.001	0.598	<0.001
Fish and seafood based dishes	40	4.5 ± 4.0	2.3	51	2.1 ± 2.4	0.4	24	8.8 ± 11.2	1.1	0.059 ± 0.022	0.062	0.010
Canned condensed soup	#	4.4 ± 5.7	0.1	45	3.9 ± 4.6	0.4	13	12.7 ± 8.6	0.4	0.762 ± 0.205	0.310	<0.001

Post-hoc analysis was not performed for food group with any group having less than 2 consumers (infant foods and infant formulae/breast milk). Groups with less than 10 consumers were excluded from the ranking (marked with #). ^1^ Percentage of participants who consumed foods in the food group; ^2^ *P*_trend_ from linear regression test for trends in medians of the three surveys; ^3^ *p* < 0.001 compared with 1995NS; ^4^ *p* < 0.001 compared with 2007NS.

**Table A4:** The contribution of the top 20 GL food groups to inter-individual variations in dietary glycaemic index (dGI) and glycaemic load (dGL) in 1995NS (*n* = 2658, all subjects included).

Food Groups	dGI			dGL		
	*β* ± SE	*Partial R*^2^	*p* Value	*β* ± SE	*Partial R*^2^	*p* Value
	Model *R*^2^ = 0.433			Model *R*^2^ = 0.912		
Bread and bread rolls	2.03 ± 0.13	0.082	<0.001	17.23 ± 0.69	0.191	<0.001
Fruit and vegetable juices	−0.14 ± 0.02	0.014	<0.001	3.21 ± 0.12	0.218	<0.001
Breakfast cereals (ready to eat)	3.01 ± 0.22	0.066	<0.001	32.73 ± 1.15	0.234	<0.001
Potatoes	1.62 ± 0.08	0.133	<0.001	6.64 ± 0.42	0.086	<0.001
Sweetened beverages	0.24 ± 0.03	0.026	<0.001	4.20 ± 0.15	0.227	<0.001
Cereal based dishes	0.34 ± 0.07	0.009	<0.001	3.96 ± 0.37	0.042	<0.001
Cake-type dessert	−0.30 ± 0.15	0.002	0.045	9.93 ± 0.78	0.058	<0.001
Flours, cereals and starches	1.06 ± 0.09	0.053	<0.001	10.67 ± 0.45	0.173	<0.001
Dairy milk	−0.44 ± 0.03	0.079	<0.001	−1.03 ± 0.15	0.017	<0.001
Sweet biscuits	0.10 ± 0.34	<0.001	0.776	9.23 ± 1.78	0.010	<0.001
Frozen milk products	−0.77 ± 0.09	0.026	<0.001	1.52 ± 0.48	0.004	0.002
Pastas	−0.93 ± 0.09	0.036	<0.001	2.45 ± 0.49	0.009	<0.001
Sugar, honey and syrups	1.75 ± 0.48	0.005	<0.001	28.88 ± 2.51	0.048	<0.001
Pome fruit	−0.93 ± 0.07	0.058	<0.001	1.51 ± 0.38	0.006	<0.001
Pastries	−0.42 ± 0.11	0.006	<0.001	−0.12 ± 0.56	<0.001	0.830
Other confectionery	3.96 ± 0.39	0.039	<0.001	46.65 ± 2.01	0.169	<0.001
Chocolates	−1.56 ± 0.30	0.010	<0.001	4.03 ± 1.58	0.002	0.011
Savory biscuits	2.30 ± 0.48	0.009	<0.001	13.79 ± 2.50	0.011	<0.001
Tropical and subtropical fruit	−0.70 ± 0.15	0.008	<0.001	4.52 ± 0.80	0.012	<0.001
Fancy breads	1.37 ± 0.31	0.007	<0.001	12.33 ± 1.62	0.022	<0.001

*β* ± SE calculated using multiple linear regression, with energy and the food groups as the predictor variables. *β* expressed as change in dGI or dGL per 100 g increase in intake of the corresponding food group.

**Table A5:** The contribution of the top 20 GL food groups to inter-individual variations in dietary glycaemic index (dGI) and glycaemic load (dGL) in 2007NS (*n* = 4828, all subjects included).

Food Groups	dGI			dGL		
	*β* ± SE	*Partial R*^2^	*p* Value	*β* ± SE	*Partial R*^2^	*p* Value
	Model *R*^2^ = 0.362			Model *R*^2^ = 0.881		
Bread and bread rolls	2.58 ± 0.12	0.084	<0.001	15.88 ± 0.55	0.149	<0.001
Breakfast cereals (ready to eat)	4.11 ± 0.18	0.094	<0.001	35.86 ± 0.82	0.284	<0.001
Fruit and vegetable juices	0.05 ± 0.03	<0.001	0.113	3.12 ± 0.14	0.093	<0.001
Potatoes	1.79 ± 0.08	0.092	<0.001	7.74 ± 0.36	0.087	<0.001
Pastas	−0.55 ± 0.06	0.015	<0.001	5.01 ± 0.29	0.060	<0.001
Flours, cereals and starches	1.65 ± 0.08	0.089	<0.001	13.60 ± 0.34	0.249	<0.001
Sweetened beverages	0.23 ± 0.03	0.016	<0.001	2.91 ± 0.12	0.115	<0.001
Dairy milk	−0.36 ± 0.03	0.034	<0.001	−0.61 ± 0.12	0.005	<0.001
Cereal based dishes	−0.01 ± 0.06	<0.001	0.819	3.91 ± 0.27	0.042	<0.001
Cake-type dessert	0.07 ± 0.16	<0.001	0.643	8.65 ± 0.69	0.031	<0.001
Fancy breads	1.60 ± 0.20	0.013	<0.001	13.96 ± 0.9	0.047	<0.001
Sweet biscuits	1.56 ± 0.32	0.005	<0.001	17.29 ± 1.43	0.029	<0.001
Savory biscuits	3.92 ± 0.39	0.020	<0.001	20.87 ± 1.75	0.029	<0.001
Other confectionery	5.20 ± 0.26	0.075	<0.001	49.42 ± 1.18	0.268	<0.001
Pome fruit	−0.86 ± 0.06	0.036	<0.001	2.11 ± 0.29	0.011	<0.001
Chocolates	−0.14 ± 0.26	<0.001	0.595	11.97 ± 1.18	0.021	<0.001
Pastries	0.11 ± 0.10	<0.001	0.264	2.67 ± 0.43	0.008	<0.001
Tropical and subtropical fruit	−0.44 ± 0.10	0.004	<0.001	3.74 ± 0.46	0.014	<0.001
Sugar, honey and syrups	2.04 ± 0.46	0.004	<0.001	32.06 ± 2.07	0.048	<0.001
Batter-based product	2.79 ± 0.22	0.033	<0.001	16.39 ± 0.97	0.056	<0.001

*β* ± SE calculated using multiple linear regression, with energy and the food groups as the predictor variables. *β* expressed as change in dGI or dGL per 100 g increase in intake of the corresponding food group.

**Table A6:** The contribution of the top 20 GL food groups to inter-individual variations in dietary glycaemic index (dGI) and glycaemic load (dGL) in 2012NS (*n* = 2374, all subjects included).

Food Groups	dGI			dGL		
	*β* ± SE	*Partial R*^2^	*p* Value	*β* ± SE	*Partial R*^2^	*p* Value
	Model *R*^2^ = 0.303			Model *R*^2^ = 0.871		
Bread and bread rolls	1.94 ± 0.19	0.042	<0.001	13.76 ± 0.81	0.108	<0.001
Cereal based dishes	0.50 ± 0.06	0.025	<0.001	4.22 ± 0.27	0.092	<0.001
Breakfast cereals (ready to eat)	3.32 ± 0.35	0.038	<0.001	28.71 ± 1.46	0.141	<0.001
Fruit and vegetable juices	−0.02 ± 0.05	<0.001	0.673	3.15 ± 0.21	0.088	<0.001
Flours, cereals and starches	2.11 ± 0.13	0.098	<0.001	16.39 ± 0.56	0.266	<0.001
Potatoes	1.61 ± 0.13	0.066	<0.001	7.40 ± 0.53	0.076	<0.001
Sweetened beverages	0.12 ± 0.03	0.005	<0.001	3.45 ± 0.14	0.201	<0.001
Cake-type dessert	−0.57 ± 0.17	0.005	<0.001	8.36 ± 0.74	0.052	<0.001
Sweet biscuits	0.12 ± 0.39	<0.001	0.752	17.85 ± 1.63	0.048	<0.001
Dairy milk	−0.57 ± 0.04	0.066	<0.001	−1.04 ± 0.19	0.013	<0.001
Savory biscuits	3.06 ± 0.41	0.023	<0.001	18.82 ± 1.75	0.047	<0.001
Pome fruit	−0.87 ± 0.09	0.041	<0.001	2.04 ± 0.37	0.013	<0.001
Fancy breads	1.32 ± 0.29	0.009	<0.001	12.81 ± 1.21	0.045	<0.001
Tropical and subtropical fruit	−0.54 ± 0.17	0.004	0.001	4.00 ± 0.70	0.013	<0.001
Cereal-, fruit-, nut-, seed-bars	2.93 ± 0.68	0.008	<0.001	20.07 ± 2.86	0.020	<0.001
Pastries	−0.68 ± 0.15	0.008	<0.001	0.16 ± 0.66	<0.001	0.811
Pastas	−0.06 ± 0.13	<0.001	0.653	3.38 ± 0.56	0.015	<0.001
Sugar, honey and syrups	3.54 ± 0.80	0.008	<0.001	42.32 ± 3.39	0.062	<0.001
Poultry based dishes	0.40 ± 0.13	0.004	0.002	0.19 ± 0.55	<0.001	0.731
Frozen milk products	−1.07 ± 0.19	0.014	<0.001	1.31 ± 0.79	0.001	0.094

*β* ± SE calculated using multiple linear regression, with energy and the food groups as the predictor variables. *β* expressed as change in dGI or dGL per 100 g increase in intake of the corresponding food group.
